# A Changing Perspective for Treatment of Chronic Kidney Disease

**DOI:** 10.3390/jcm10173840

**Published:** 2021-08-27

**Authors:** Giacomo Garibotto

**Affiliations:** Department of Internal Medicine, University of Genoa, 16128 Genova, Italy; gari@unige.it; Tel.: +39-(0)103538989

**Keywords:** chronic kidney disease, CKD–MBD, hyperkalemia, vascular calcification

Chronic kidney disease (CKD) is now an enormous worldwide health problem. The incidence and prevalence of CKD is high and still increasing, mainly owing to obesity and diabetes, and is associated with growing morbidity and mortality [1]. Overall CKD mortality has increased by ˜32% over the last 10 years, making it one of the fastest-rising major causes of death, together with diabetes and dementia [2]. CKD is the 12th most common primary cause of death, accounting for about 1 million deaths per year worldwide [1]. CKD and end-stage renal disease (ESRD) are characterized by the progressive development of a series of complications, such as hypertension, left-ventricular hypertrophy [LVH] anemia, hyperkalemia, hypervolemia, hyperphosphatemia with mineral and bone disorders (CKD–MBD), metabolic acidosis, hyperuricemia and wasting; all of these complications have been shown to be associated with adverse outcomes, and can contribute either individually or in association to the cardiovascular morbidity and mortality observed in CKD. 

Current management of CKD includes blood pressure control, treatment of albuminuria with angiotensin-converting enzyme inhibitors or angiotensin II receptor blockers, nutritional intervention, avoidance of potential nephrotoxins and obesity, drug dosing adjustments, and cardiovascular risk reduction. Recent progress in our understanding of CKD pathophysiology together with the development of novel therapeutic agents has led to a renewed attention on the treatment of CKD and its associated metabolic complications which are now amenable to intervention. It is this point that led to the creation of this relevant Special Issue. I am happy to know that scientists from several parts of the world responded with enthusiasm to the opportunity, sending papers which involve genetics, pathophysiology and epidemiology together with the novel therapeutical approaches to CKD.

Genome-wide association studies have identified hundreds of loci where genetic variants are associated with CKD; however, more than 90% of these variants are in noncoding regions of the genome and how they cause disease is still unclear. Lysosomal beta-d-mannosidase (*MANBA*) is an exoglycosidase involved in the sequential degradation of the N-glycosylproteins glycans. Recently, the *MANBA* gene was proposed as a kidney disease severity gene. In this issue, Hye-Rim Kim et al. [3] evaluated, by integrating CKD-related variants and kidney expression quantitative trait loci (eQTL) data, the effects of *MANBA* gene variants on CKD and kidney function-related traits in the Korean Genome and Epidemiology Study (KoGES) cohort. Their study observed 20 single nucleotide polymorphisms (SNPs) that showed a statistically significant association with CKD and kidney function-related traits among 229 SNPs of the MANBA gene. In addition, rs4496586, which had the highest significance for CKD, was associated with MANBA gene expression in renal tubules and glomeruli. In conclusion, this study strongly suggests that *MANBA* gene variants are associated with CKD and kidney function-related traits. 

Even if obesity and diabetes largely contribute to the CKD epidemics, their interaction with kidney toxins is incompletely understood. Hagedoorn et al. [4] investigated if lifestyle-related exposures (diet and smoking) contribute to blood cadmium and lead concentrations, as well as whether they are associated with the prevalence of diabetic kidney disease (DKD). In a cross-sectional analysis of a cohort of 231 patients with type 2 diabetes included in the DIAbetes and LifEstyle Cohort Twente (DIALECT-1), median cadmium and lead blood concentrations were below acute toxicity values [2.94 nmol/L for cadmium and 0.07 µmol/L for lead, respectively]. However, every doubling of lead concentration was associated with a 1.75 (95% confidence interval [CI]: 1.11–2.74) times higher risk for albuminuria. In addition, both cadmium and lead were associated with an increased risk for reduced creatinine clearance. The association between cadmium and lead and the prevalence of DKD suggests that they might contribute to the development of this major diabetes complication.

It is still challenging to predict acute and chronic kidney injury after kidney surgery. This is mainly due to a scarcity in sensitive and specific biomarkers for predicting CKD progression early. The development of ESRD may take several years, therefore surrogate endpoints such as albuminuria and serum creatinine have been increasingly used in trials over hard endpoints to predict CKD progression. Acute kidney injury (AKI) is a common complication after nephrectomy in renal cell cancer, with radical nephrectomy being associated with a higher risk than partial nephrectomy. Several predictive factors for CKD following radical nephrectomy (RN) or partial nephrectomy (PN) have been identified. However, the association between AKI and long-term renal function after radical nephrectomy has not been evaluated fully. Won Ho Kim et al. [5], in a retrospective study of 558 cases of radical nephrectomy (median follow-up of 35 months), observed that AKI occurred in 43.2% of cases, and, more importantly, CKD3a developed in 40.5% of patients. The incidence of new-onset CKD was significantly higher in patients with AKI than those without at all follow-up time points after surgery. In conclusion, their analysis demonstrated a strong association between AKI after radical nephrectomy and long-term renal functional deterioration.

Preservation of kidney function can improve outcomes and can be achieved through non-pharmacological strategies and CKD-targeted pharmacological interventions. Gout as well as asymptomatic hyperuricemia have been associated with several traditional cardiovascular risk factors both in adults and children with CKD [6]. In vitro studies and animal models support a role for uric acid mediating both hemodynamic and tissue toxicity leading to glomerular and tubule-interstitial damage, respectively. Nevertheless, two recent well-designed trials failed to show any benefit of allopurinol treatment on renal outcomes, casting doubts on the expectations of renal protection by the use of urate lowering treatment. In addition, a trend for increased mortality was observed in the interventional arms. Russo et al. [7] critically reviewed results from all available randomized controlled trials comparing a urate-lowering agent with placebo or no study medication for at least 12 months. According to the authors, the analysis of the literature does seem to leave it open to the possibility of demonstrating the beneficial effect of urate-lowering agents in future trials. In consideration that both vascular and kidney damages induced by uric acid cannot regress once they have been established, patients with better-preserved renal function and children might benefit more from an early treatment. Adequately powered, randomized, placebo-controlled trials with appropriate selection criteria are needed to determine whether specific patient groups could benefit from urate-lowering agents.

Early identification of the risk factors for CKD and its progression is critical for prevention of kidney damage and adverse outcomes. Cheng-Sheng Yu et al. [8] applied the clustering heatmap and random forest methods to provide an interactive visualization of patients with different CKD stages in a retrospective cohort study. They observed that an index of body composition (waist circumference) and a few biochemical parameters (uric acid, blood urea nitrogen, serum glutamic oxaloacetic transaminase, and HbA1c) were significantly associated with CKD. In their analysis, CKD was associated with obesity, hyperglycemia, and liver function. Interestingly, hypertension and HbA1c levels were associated in the same cluster with a similar pattern. Despite the study limitations (inherent to its retrospective, cross-sectional cohort approach), their data suggest that the clustering heatmap may provide a new predictive model for a high risk of rapid CKD progression.

Continuous growth in the incidence of DKD is the main driver of CKD burden [1,2]. DKD is a major cause of morbidity and mortality in diabetes [9]. Despite advances in the nephroprotective treatment of diabetes, DKD remains the most common complication, driving the need for renal replacement therapies [RRT] worldwide, and its incidence is increasing. Until recently, prevention of DKD progression was based around strict blood pressure [BP] control, using renin–angiotensin system blockers that simultaneously reduce BP and proteinuria, adequate glycemic control and control of cardiovascular risk factors. New drugs which modify intrarenal haemodynamics (such as renin–angiotensin–aldosterone pathway modulators and SGLT2 inhibitors) can preserve the kidney from damage by decreasing intraglomerular pressure independently of blood pressure and glucose control, whereas other novel agents (such as mineralocorticoid receptor antagonists) might offer kidney protection through their antifibrotic mechanisms in DKD. In this issue, Gorriz et al. [10] review the potential of Glucagon-like peptide-1 Receptor Agonist (GLP-1RA) for adequate glycemic control in multiple stages of DKD without increasing risk of hypoglycemia; in addition, GLP-1RA may prevent the onset of macroalbuminuria and slow the decline of glomerular filtration rate (GFR) in diabetic patients, also offering additional benefit in weight reduction, cardiovascular and other kidney outcomes. Trials to assess the impact of GLP-1RA treatments on primary kidney endpoints in DKD are ongoing and some of them will be soon available. 

Cardiovascular [CV] disease is a leading cause of morbidity and mortality in patients with CKD and in those on hemodialysis [HD] [11] and is strongly associated with atherosclerosis and vascular calcification [VC]. Patients with CKD have a higher prevalence of vascular calcifications as renal function declines, which will result in increased mortality. Serum calciprotein particles [CPPs] are colloidal nanoparticles that have a prominent role in the initiation and progression of VC. In this issue, Silaghi et al. [12] reviewed the usefulness of the T_50_ test, a novel test that measures the conversion of primary to secondary CPPs, indicating the tendency of serum to calcify, in the assessment of VC. They also made a comprehensive review of the regulation of serum CPP levels, and explored the effects of CPPs and calcification propensity on outcomes. In addition, new topics were raised regarding possible clinical uses of T_50_ in the assessment of VC, particularly in patients with CKD, including possible opportunities in VC management.

Hyperphosphatemia is a common complication of CKD. Even if severe hyperphosphatemia is clinically asymptomatic, it is associated with morbidity and poor outcome. Hyperphosphatemia is an emerging cardiovascular risk factor in CKD, by contributing to vascular calcification. However, mechanisms by which hyperphosphatemia is associated to CV complications are not clearly understood. Abbasian et al. [13] studied the effects of phosphate [Pi] on the release of pro-coagulant activity from endothelial microvesicles [MV] in male Sprague–Dawley rats with experimental CKD; rodents were randomly allocated to receiving high [1.2%] or low [0.2%] dietary phosphorus; and sham-operated controls receiving high [1.2%] phosphorus. After 14 days, as compared to sham controls, high-phosphorus CKD rats presented elevated total plasma MVs, expressed higher CD144 (a major component of endothelial adherens junctions which is expressed by endothelial cells during development), and enhanced procoagulant activity. The results observed in the rat model by Abbasian et al. [13] show that hyperphosphatemia may induce an increase in circulating pro-coagulant MVs, suggesting an important link between elevated circulating phosphate and thrombotic risk in CKD. 

Both in CKD and HD patients, volume and pressure overload ultimately lead to LVH, a significant predictor of increased CV events [14]. Although many studies have used pre-HD blood pressure (BP) to determine optimal BP levels, the optimal timing and measurement techniques of blood pressure in HD patients are not well established. In a prospective observational study, Hiroaki et al. [15] aimed at identifying the ideal timing and setting for measuring BP; they observed an association between increased CV events and LVMI > 156 g/m^2^ in patients with diabetes mellitus, after performing multivariate regression analysis. In addition, they found that pre-HDBP at the start of the week, post-HDBP at the end of the week, and weekly averaged BP (WABP) were independently associated with LVMI on univariate regression analysis of follow-up. Multiple BP measurements taken before, during and after dialysis were confirmed to be the most accurate assessment format, a finding which suggests that multiple measurements of BP should be performed and then averaged in this clinical setting.

Hyperkalemia is a very common CKD complication, which accounts for a large number of urgent visits in emergency departments, as well as high mortality. Hyperkalemia is commonly observed in patients with chronic heart failure [CHF], in CKD stage 3–5, and in patients with diabetes mellitus. Among CKD patients, those requiring dialysis represent a group at particularly high risk of hyperkalemia. For a long time, the only therapeutic option for increasing fecal K^+^ excretion has been represented by sodium polystyrene sulfonate, a cation-exchanging resin. Recently, new drugs able to promote gastrointestinal potassium elimination, namely patiromer and sodium zirconium cyclosilicate, have been developed and studied in large trials, proving their efficacy and safety in different clinical contexts. In this review, Esposito et al. [16] have reviewed the pathophysiology of hyperkalemia, focusing on the mechanisms of action and the clinical data of patiromer and sodium zirconium cyclosilicate, considering that these new treatments may represent a chance to improve the management of both acute and chronic hyperkalemia.

Assessing cognitive, nutritional and functional status in elderly subjects with CKD is emerging as a new tool to stratify the risk to develop ESRD and death [17]. In addition, the prognostic evaluation of older adults with CKD is crucial to identify the most appropriate clinical decision-making process for patients and their families. A multidimensional assessment (MPI) may represent an important aspect in predicting short- and long-term all-cause mortality in elderly patients with CKD [18]. In particular, in CKD patients, the MPI was shown to be more accurate in predicting mortality when compared to the eGFR alone [18]. In this issue, Lai et al. [19] longitudinally studied the associations in MPI, both the hospitalization and mortality in clinically stable CKD (*n* = 105) patients on conservative therapy (eGFR ≤ 60 mL/min, stage 3–5 KDOQI), or renal replacement therapy (HD = 32 pts or PD = 36 pts), for at least 3 months. A total of 173 patients, with a median age of 76 years, was studied. The median duration of all the hospitalizations was 6 days and the number of deaths was 33. MPI significantly correlated with days and number of hospitalizations per year. According to the findings, MPI was associated with outcomes in patients with renal disease, suggesting that a multidimensional evaluation should be implemented in this clinical context.

Many of the important issues dealing on prevention and treatment of CKD are addressed in this volume. We thank the writers, the large number of investigators and the MDPI staff for their leadership in producing this Special Issue. We hope the readers will enjoy and benefit from new insight.

## Data Availability

Not applicable.
